# Psychometric Validation of the Korean Version of the Professional Bereavement Scale Among Tertiary Hospital Nurses

**DOI:** 10.1155/jonm/1180779

**Published:** 2026-08-06

**Authors:** Haeyoung Lee, Sun Joo Jang, Sophia J. Chung

**Affiliations:** ^1^ Red Cross College of Nursing, Chung-Ang University, Seoul, South Korea, cau.ac.kr; ^2^ College of Nursing & Research Institute of Nursing Science, Seoul National University, Seoul, South Korea, snu.ac.kr

## Abstract

**Background:**

Nurses experience professional bereavement—distinct grief reactions following patient deaths that differ fundamentally from familial bereavement; however, existing measurement tools lack specificity for assessing these unique professional experiences.

**Purpose:**

This study aimed to validate the Korean version of the Professional Bereavement Scale, which comprises two subscales: Short‐term bereavement reactions (SBR) and accumulated global changes (AGC).

**Methods:**

Data from 340 nurses working for at least one month in tertiary hospitals in Korea were used in this study. The content, construct, convergent, discriminant, and criterion validities were evaluated. Cronbach’s *α* and intraclass correlation coefficients were also evaluated to determine reliability.

**Results:**

Three factors with 17 items were identified for SBR, whereas three factors with seven items were identified for AGC. The validity and reliability of the scale were acceptable. Cronbach’s *α* was 0.91 for SBR and 0.65 for AGC. The intraclass correlation coefficients for SBR and AGC were 0.83 and 0.81, respectively.

**Conclusions:**

This study evaluated the psychometric properties of the Korean version of the Professional Bereavement Scale; the SBR was found to be a valid and reliable tool, whereas the AGC requires further validation.

**Implications for Nursing Management:**

The Korean version of the Professional Bereavement Scale was validated in this study. This enables nurse managers to systematically assess professional bereavement among nurses in a Korean context, thereby facilitating the early identification of grieving nurses and the development of targeted support interventions to improve their well‐being and reduce burnout.

## 1. Introduction

Nurses are committed to delivering optimal care and fulfilling complex professional roles in end‐of‐life care and during the dying process. However, such involvement inevitably entails a significant emotional burden [1]. Repeated exposure to patient death in nursing practice is increasingly conceptualized as professional bereavement, referring to the emotional and professional reactions that healthcare professionals experience following the death of a patient [2]. Although providing end‐of‐life care and accompanying patients through the dying process constitute a fundamental component of the professional role of nurses [3], the death of patients in their care often evokes profound emotional distress [4]. Such experiences compromise the personal well‐being and working conditions of nurses, potentially yielding prolonged adverse effects if left unaddressed [5].

Moreover, nurses frequently report that they have not received adequate preparation or training to deal with patient death and thus experience considerable emotional strain when confronted with such events [6, 7]. Notably, Zheng et al. [7] emphasized that the encounters of newly graduated nurses with patient death at the outset of their careers represent a harsh clinical reality and a formidable emotional challenge. Consequently, they called for heightened organizational awareness of this issue and recommended the provision of targeted support, including specialized educational opportunities, to enable novice nurses to cope more effectively with bereavement in clinical practice.

Vázquez‐Sánchez et al. [5] reported that nurses who experienced patient death during the COVID‐19 pandemic exhibited symptoms of professional grief along with related cognitive reactions, emphasizing the importance of recognizing professional bereavement and providing appropriate coping strategies and support. In this context, the negative preconceptions and attitudes of nurses toward death may generate considerable stress and frustration during the patient’s dying process, which in turn can hinder the dignified death of the patient. Therefore, understanding the attitudes and responses of nurses during the dying process is critical [4].

Professional bereavement encompasses immediate and transient physical and emotional responses, as well as accumulated changes across various aspects of personal and professional life resulting from repeated exposure to patient deaths [2, 8]. Notably, professional bereavement should be clearly distinguished from familial bereavement, and instruments designed to measure professional bereavement should not merely represent a scaled‐down version of tools originally developed for familial bereavement [8].

Several instruments have been used to assess bereavement‐related experiences, such as the Grief Experience Inventory [9], Inventory of Complicated Grief [10], and Texas Revised Inventory of Grief [11]. However, these measures were originally developed to assess familial or individual bereavement and were not designed to capture the professional and contextual dimensions of bereavement experienced by healthcare professionals, thereby limiting their applicability in professional settings [12]. Although the Adult Oncologists Grief Questionnaire [13] incorporates a professional standpoint, it has several limitations. For instance, it was developed without sufficient validation of reliability and validity, was restricted to physicians treating patients with cancer, and did not account for the long‐term consequences of bereavement.

Furthermore, using a scoping review, Chen et al. [14] pointed out that professional bereavement has been implicitly regarded as equivalent to familial bereavement in academic discourse and stressed the necessity of establishing a clear definition of professional bereavement as well as developing an appropriate measurement tool. Consequently, Chen and Chow [12] developed the Professional Bereavement Scale (PBS), designed to capture short‐term bereavement reactions (SBR) and accumulated global changes (AGC). However, despite the increasing recognition of professional bereavement and the growing need for its measurement, there remains a lack of validated tools that adequately capture this construct across diverse cultural and clinical contexts.

A scoping review examining grief experiences among nurses and physicians highlighted that frontline healthcare professionals face a considerable cumulative emotional burden related to repeated patient deaths [15]. In particular, nurses working in tertiary hospitals are frequently exposed to high‐acuity patients, complex end‐of‐life care, and repeated patient deaths. Previous research has demonstrated that exposure to patient death evokes significant emotional burden and professional bereavement among nurses [4, 6]. Given nurses’ continuous bedside presence and sustained caregiving roles, they may be particularly vulnerable to intensified grief‐related burden in clinical practice.

In the healthcare system, tertiary hospitals provide highly specialized care, and nurses in these settings often establish sustained professional relationships with patients and families. Despite these distinctive clinical characteristics, validated instruments specifically designed to assess professional bereavement among tertiary hospital nurses remain limited. This gap highlights the necessity of rigorously translating and validating an appropriate scale to ensure accurate assessment and to inform targeted interventions. Accordingly, this study aimed to evaluate the reliability and validity of the Korean version of the PBS among tertiary hospital nurses in Korea who had experienced the death of patients with whom they had established sustained professional relationships during end‐of‐life care.

## 2. Materials and Methods

### 2.1. Study Design

This study was a secondary data analysis with a methodological design to evaluate the validity and reliability of the Korean version of the PBS [12].

## 3. Participants and Data Collection

Data from 340 nurses who met the inclusion criteria of a parent study [16] conducted between July and August 2023 were used. The sample size in this study was adequate because a sample size of at least 300 participants is recommended for confirmatory factor analysis (CFA) [17]. The inclusion criteria for this study were nurses (1) who had been working for at least one month in tertiary hospitals in Korea, (2) who voluntarily agreed to participate, (3) who had no difficulties completing an online survey, and (4) who had experienced the death of a patient within the past year. The exclusion criteria were nurses (1) who were not engaged in nursing care and (2) who were diagnosed with psychiatric diseases.

### 3.1. Ethical Considerations

This study used the existing data and was exempted from review by the institutional review board of Chung‐Ang University (no. 1041078‐20230421‐HR‐112; approved on June 9, 2023). In the parent study, informed consent was obtained from all participants before data collection [16]. Thus, informed consent was waived for this secondary data analysis.

### 3.2. Measures

#### 3.2.1. PBS

The PBS is an instrument for measuring professional bereavement experiences and consists of 32 items rated on a five‐point Likert scale with two subscales: SBR and AGC [12]. The 17‐item PBS–SBR comprises four factors: frustration and trauma, guilt, grief, and being moved. The 15‐item PBS–AGC consists of five factors: new insights, more acceptance of limitations, more death‐related anxiety, less influence from patient deaths, and better coping with patient deaths [12].

At the time of development, the Comparative Fit Index (CFI) = 0.94, root mean square error of approximation (RMSEA) = 0.09, standardized root mean square residual (sRMR) = 0.05, and all factor loadings for the PBS–SBR were > 0.50. Cronbach’s *α* for each factor ranged from 0.89 to 0.96. For the PBS–AGC, CFI = 0.96, RMSEA = 0.08, sRMR = 0.05, and all factor loadings were > 0.50. Cronbach’s *α* ranged from 0.86 to 0.95 for the PBS–AGC at the time of development [12].

#### 3.2.2. Professional Quality of Life (ProQOL)

ProQOL is “the quality one feels in relation to their work as a helper” [18]. The ProQOL Scale measures positive and negative aspects and comprises three subscales: compassion satisfaction, secondary traumatic stress, and burnout [18]. Compassion satisfaction is the pleasure of helping others or doing work well [18]. Secondary traumatic stress is related to work‐related exposure to extremely stressful events [18]. Burnout is an accumulated feeling of hopelessness and difficulty in doing work effectively [18]. Each subscale has 10 items scored on a five‐point Likert scale (1 = “never” and 5 = “very often”), with scores ranging from 10 to 50.

At the time of development [18], Cronbach’s *α*s for compassion satisfaction, secondary traumatic stress, and burnout were 0.88, 0.81, and 0.75, respectively. In this study, Cronbach’s *α*s for compassion satisfaction, secondary traumatic stress, and burnout were 0.88, 0.85, and 0.70, respectively.

### 3.3. Verification Process of the Preliminary Tool

After obtaining permission from the original developers of the PBS, we translated the PBS into Korean. The expert panel comprising a total of 8 experts from diverse nursing fields, including geriatric, psychiatric, and critical care nursing, reviewed the translated items. After several agreement stages to confirm the accuracy of the translation and appropriateness of the expression, a preliminary Korean version was finalized. Reverse translation was performed by a nursing professor who was proficient in English and Korean and had never seen the original English tools. We then compared the original English and reverse‐translated versions and confirmed the final Korean version of the PBS.

### 3.4. Data Analysis

Data were analyzed using SPSS Statistics software Version 30.0 and AMOS 30.0 (IBM Corp., Armonk, NY, United States). A content validity index (CVI) was calculated to assess the validity of the tool. The Kaiser–Meyer–Olkin (KMO) and Bartlett’s tests of sphericity were performed to determine whether the data collected were suitable for factor analysis. Exploratory factor analysis (EFA) and CFA were performed to confirm the construct validity. The following model fit was used for model fitness: χ^2^, χ^2^/df, Normed Fit Index (NFI), Tucker–Lewis Index (TLI), CFI, RMSEA, and sRMR. Standardized factor loading (*β*), average variance extracted (AVE), and construct reliability (CR) were confirmed to verify the convergent validity. Discriminant validity was assessed using the heterotrait–monotrait (HTMT) ratio [19]. According to a previous study on the development and validation of the PBS [12], criterion validity of the PBS–SBR was confirmed through the correlation with burnout from the ProQOL, and the PBS–AGC through compassion satisfaction and secondary traumatic stress. Cronbach’s *α* was used to verify the internal consistency, and test–retest reliability was confirmed through the intraclass correlation coefficient (ICC) calculated by retesting 49 participants at an interval of two weeks. The significance level for all analyses was < 0.05.

## 4. Results

### 4.1. General Characteristics of Participants

The mean age of the participants was 31.89 years (standard deviation [SD]  5.36). 314 (92.4%) participants were female, 223 (66.2%) were unmarried (including separated and widowed), 187 (55.0%) were religious, and 279 (82.1%) had a bachelor’s degree. The average number of total working years was 83.23 months (SD 57.45). The average number of expired patients in current units within a month was 7.17 (SD 9.73) (Table 1).

**TABLE 1:** General characteristics of the participants (*N* = 340).

Characteristics	Categories	*n* (%)	M (SD)
Age (years)			31.89 (5.36)

Sex	Female	314 (92.4)	
	Male	26 (7.6)	

Marital status	Unmarried	223 (66.2)	
	Married	115 (33.8)	

Religion	No	153 (45.0)	
	Yes	187 (55.0)	

Educational level	3‐year college	28 (8.2)	
	Bachelor’s degree	279 (82.1)	
	Above master’s degree	33 (9.7)	

Subjective health status	Poor	38 (11.2)	
	Normal	116 (34.1)	
	Good	186 (54.7)	

Total working experience (month)			83.23 (57.45)

Average number of expired patients within a month			7.17 (9.73)

### 4.2. Item Analysis

#### 4.2.1. PBS–SBR

The mean score for each item was 1.42–2.75. The skewness ranged from −0.73 to 0.44, while the kurtosis ranged from −1.10 to 0.33, indicating that the items were all normally distributed based on the criteria of Kline [20], with an absolute skewness value of < 3 and an absolute kurtosis value of < 10. Item‐total correlation coefficients (ITC‐r) ranged from 0.35 to 0.75, confirming the internal consistency. Cronbach’s *α* if an item was deleted ranged from 0.90 to 0.91 (Table 2).

**TABLE 2:** Item analysis for the Korean version of the Professional Bereavement Scale.

Items	M	SD	Min	Max	Skewness	Kurtosis	ITC‐r	*α* if item deleted
Short‐term bereavement reaction								
1. I felt sad	2.75	0.91	0	4	−0.73	0.33	0.56	0.91
2. I felt grief	2.28	1.11	0	4	−0.32	−0.71	0.60	0.90
3. I felt that life is uncertain	2.69	1.10	0	4	−0.67	−0.21	0.49	0.91
4. I was moved by the patient’s family’s understanding	2.49	0.97	0	4	−0.46	−0.17	0.38	0.91
5. I felt fatigue	2.63	0.99	0	4	−0.65	0.10	0.53	0.91
6. I was moved by the patient’s family’s gratitude	2.51	0.96	0	4	−0.47	−0.12	0.35	0.91
7. I blamed myself	1.50	1.13	0	4	0.39	−0.72	0.64	0.90
8. I felt pity for the death of the patient	2.69	0.98	0	4	−0.61	−0.08	0.53	0.91
9. I felt guilty	1.74	1.18	0	4	0.14	−0.94	0.72	0.90
10. I thought that I am not a good nurse	1.59	1.11	0	4	0.24	−0.73	0.60	0.90
11. I was confused about why the patient died	1.42	1.16	0	4	0.44	−0.84	0.62	0.90
12. I felt nervous and worried about potential professional‐patient conflicts	1.78	1.21	0	4	0.07	−1.10	0.51	0.91
13. I doubted the value of my occupation	1.55	1.13	0	4	0.34	−0.75	0.63	0.90
14. I felt exhausted	1.55	1.19	0	4	0.22	−1.03	0.61	0.90
15. I felt frustrated	1.59	1.20	0	4	0.20	−1.04	0.75	0.90
16. The scene of the event intruded on my mind repeatedly	2.04	1.21	0	4	−0.21	−0.96	0.69	0.90
17. I felt anxious for my own death in the future	2.01	1.21	0	4	−0.24	−1.01	0.62	0.90
Accumulated global changes								
1. I am more aware that death is inevitable	3.15	0.85	0	4	−1.12	1.47	0.32	0.72
2. I am more aware that life is uncertain	3.05	0.86	0	4	−0.93	1.02	0.24	0.73
3. I cherish my life more	2.96	0.82	0	4	−0.48	−0.27	0.33	0.72
4. I feel fatigued by my job	2.68	0.92	0	4	−0.41	−0.41	0.33	0.72
5. I am more anxious about the future deaths of my loved ones	2.42	1.06	0	4	−0.50	−0.44	0.19	0.74
6. The immediate impact that a patient death has on me becomes weaker	2.17	1.02	0	4	0.05	−0.76	0.37	0.72
7. The after‐effects of patient deaths become weaker for me	2.28	1.04	0	4	−0.03	−0.86	0.44	0.71
8. I cherish the present more	2.82	0.80	0	4	−0.66	0.74	0.32	0.72
9. I am better at coping with patient deaths	2.41	1.03	0	4	−0.32	−0.60	0.45	0.71
10. I achieve more acceptance of patient deaths	2.52	1.04	0	4	−0.41	−0.46	0.41	0.71
11. I deliberately avoid building very close relationships with patients	1.91	1.12	0	4	−0.11	−0.98	0.30	0.73
12. I am more aware of the limitation of medical science	2.78	0.91	0	4	−0.79	0.65	0.46	0.71
13. The goals in my career have become more practical	2.80	0.88	0	4	−0.62	0.06	0.41	0.71
14. I achieve more acceptance of my own death	2.63	0.92	0	4	−0.68	0.31	0.40	0.72
15. I am more anxious about my own mortality	1.78	1.05	0	4	−0.02	−0.89	0.12	0.75

*Note: M*, mean; SD, standard deviation, ITC, item‐total correlation.

#### 4.2.2. PBS–AGC

The mean score for each item was 1.78–3.15. The skewness and the kurtosis ranged from −1.12 to 0.05 and from −0.98 to 1.47, respectively, meeting Kline’s normal distribution criteria [20]. ITC‐r ranged from 0.12 to 0.46. Cronbach’s *α* if an item was deleted ranged from 0.71 to 0.75 (Table 2). Although Items 2, 5, and 15 had ITC‐r below 0.30, deleting them did not increase Cronbach’s *α*; thus, all items were retained.

### 4.3. Reliability Verification

With the data from 340 nurses, Cronbach’s *α* of the PBS–SBR was 0.91. Cronbach’s *α* for Factors 1, 2, and 3 were 0.89, 0.84, and 0.72, respectively. The ICC after a two‐week retest interval was 0.83. Cronbach’s *α* of the PBS–AGC was 0.65. Cronbach’s *α* for Factors 1, 2, and 3 were 0.76, 0.66, and 0.66, respectively. The ICC for test–retest reliability was 0.81.

### 4.4. Validity Analyses

#### 4.4.1. Content Analysis

An expert panel comprising eight nursing professors and clinical experts evaluated the content validity of the Korean version of the PBS. The Item CVI ranged from 0.75 to 1.00, and the scale CVI was 0.98.

#### 4.4.2. Construct Validity

An EFA was conducted to confirm the construct validity of the Korean version of the PBS using a random subsample of 179 participants from the total pool of 340. Principal components and varimax orthogonal rotations were applied. Eigenvalues of ≥ 1 and scree plots were used to extract the factors. Additional detailed information for the PBS–SBR and PBS–AGC is provided in Tables 3 and 4, respectively.

**TABLE 3:** Factor loadings for the Korean version of the short‐term bereavement reactions from the professional bereavement scale.

Items	Factor 1	Factor 2	Factor 3
	Factor loading		
Factor 1. Guilty and frustration			
7. I blamed myself	0.73		
9. I felt guilty	0.72		
10. I thought that I am not a good nurse	0.76		
11. I was confused about why the patient died	0.78		
12. I felt nervous and worried about potential professional–patient conflicts	0.54		
13. I doubted the value of my occupation	0.83		
14. I felt exhausted	0.67		
15. I felt frustrated	0.71		
Factor 2. Grief and trauma			
1. I felt sad		0.63	
2. I felt grief		0.69	
3. I felt that life is uncertain		0.71	
5. I felt fatigue		0.58	
8. I felt pity for the death of the patient		0.62	
16. The scene of the event intruded on my mind repeatedly		0.58	
17. I felt anxious for my own death in the future		0.58	
Factor 3. Being moved			
4. I was moved by the patient’s family’s understanding			0.83
6. I was moved by the patient’s family’s gratitude			0.82
Eigenvalue	4.93	3.48	1.96
Explained variance (%)	29.00	20.48	11.52
Cumulative variance (%)	29.00	49.49	61.01

*Note:* Kaiser–Meyer–Olkin measure for sampling adequacy: 0.91; Bartlett’s sphericity test: χ^2^ = 1529.02, *p* < 0.001.

**TABLE 4:** Factor loadings for the Korean version of the accumulated global changes from the professional bereavement scale.

Items	Factor1	Factor2	Factor3
	Factor loading		
Factor 1. More acceptance of death			
7. The after‐effects of patient deaths become weaker for me	0.75		
9. I am better at coping with patient deaths	0.85		
10. I achieve more acceptance of patient deaths	0.84		
Factor 2. Cherishing the moments			
3. I cherish my life more		0.86	
8. I cherish the present more		0.86	
Factor 3. New insight into life and death			
1. I am more aware that death is inevitable			0.82
2. I am more aware that life is uncertain			0.87
Eigenvalue	2.03	1.62	1.44
Explained variance (%)	28.96	23.16	20.56
Cumulative variance (%)	28.96	52.12	72.69

*Note:* Kaiser–Meyer–Olkin measure for sampling adequacy: 0.59; Bartlett’s sphericity test: χ^2^ = 291.95, *p* < 0.001.

The KMO value was 0.91 for the PBS–SBR, and the results of Bartlett’s test of sphericity showed χ^2^ = 1529.02 (*p* < 0.001), indicating the data were suitable for factor analysis. Three factors were extracted based on the eigenvalues and scree plot. The communality ranged from 0.35 to 0.72. The factor loadings ranged from 0.54 to 0.83. The eigenvalues for Factors 1, 2, and 3 were 4.57, 3.84, and 1.91, respectively. The explained variances were 29.00%, 20.48%, and 11.52% for Factors 1, 2, and 3, respectively. The cumulative explained variance was 61.01%.

Three factors were extracted from the EFA that differed from the four factors in the original tool. Four items from Factor 1 (frustration and trauma) and all items from Factor 2 (guilt) from the original version were grouped into Factor 1 in the Korean version. Three items from Factor 1 and all four items from Factor 3 (grief) were grouped into Factor 2. Finally, in this study, Factor 1 was labeled as “Guilty and Frustration,” Factor 2 as “Grief and Trauma,” and Factor 3 as “Being Moved.”

The results of the model fit indices from CFA with the remaining 161 cases were as follows: χ^2^ = 315.73 (*p* < 0.001), χ^2^/df = 2.72, NFI = 0.77, TLI = 0.81, CFI = 0.84, RMSEA = 0.10, and sRMR = 0.07. Paths exhibiting a modification index of ≥ 10 and similarities in item wording or related content were identified to improve model fitness. The results of the modified model (Model 2) after establishing the correlations between the errors were as follows: χ^2^ = 221.49 (*p* < 0.001), χ^2^/df = 2.01, NFI = 0.84, TLI = 0.90, CFI = 0.91, RMSEA = 0.08, and sRMR = 0.06 (Table 5).

**TABLE 5:** Confirmatory factor analysis of the Korean version of the Professional Bereavement Scale (*n* = 161).

Factors	Items	Standardized estimate	Critical ratio (*p*)	AVE	CR	HTMT		
						F1‐F2	F1–F3	F2‐F3
Short‐term bereavement reaction								
1	7	0.66	—	0.47	0.88	0.82	0.49	—
	9	0.75	9.64 (< 0.001)					
	10	0.73	7.87 (< 0.001)					
	11	0.66	7.28 (< 0.001)					
	12	0.54	6.05 (< 0.001)					
	13	0.76	8.13 (< 0.001)					
	14	0.59	6.83 (< 0.001)					
	15	0.78	8.41 (< 0.001)					
2	1	0.72	—	0.42	0.83	0.82	—	0.42
	2	0.80	7.23 (< 0.001)					
	3	0.60	6.32 (< 0.001)					
	5	0.53	5.84 (< 0.001)					
	8	0.57	6.05 (< 0.001)					
	16	0.70	7.23 (< 0.001)					
	17	0.56	6.22 (< 0.001)					
3	4	0.77	—	0.57	0.73	—	0.49	0.42
	6	0.73	5.09 (< 0.001)					
Model 1: χ^2^ = 315.73 (*p* < 0.001), χ^2^/df = 2.72, NFI = 0.77, TLI = 0.81, CFI = 0.84, RMSEA = 0.10, standardized RMR = 0.07								
Model 2: χ^2^ = 221.49 (*p* < 0.001), χ^2^/df = 2.01, NFI = 0.84, TLI = 0.90, CFI = 0.91, RMSEA = 0.08, standardized RMR = 0.06								
Accumulated global changes								
1	7	0.48		0.58	0.79	0.32	0.15	—
	9	0.88	5.77 (< 0.001)					
	10	0.85	5.87 (< 0.001)					
2	3	0.74		0.45	0.62	0.32	—	0.46
	8	0.59	3.75 (< 0.001)					
3	1	0.90		0.60	0.74	—	0.15	0.46
	2	0.62	3.47 (< 0.001)					
Model fit index: χ^2^ = 19.51 (*p* = 0.052), χ^2^/df = 1.77, NFI = 0.95, TLI = 0.97, CFI = 0.98, RMSEA = 0.05, standardized RMR = 0.03								

Abbreviations: AVE, average variance extracted; CFI, Comparative Fit Index; CR, composite reliability; HTMT, heterotrait–monotrait ratio; NFI, Normed Fit Index; RMSEA, root mean square error of approximation; Standardized RMR, standardized root mean square residual; TLI, Tucker–Lewis Index.

**FIGURE 1:**
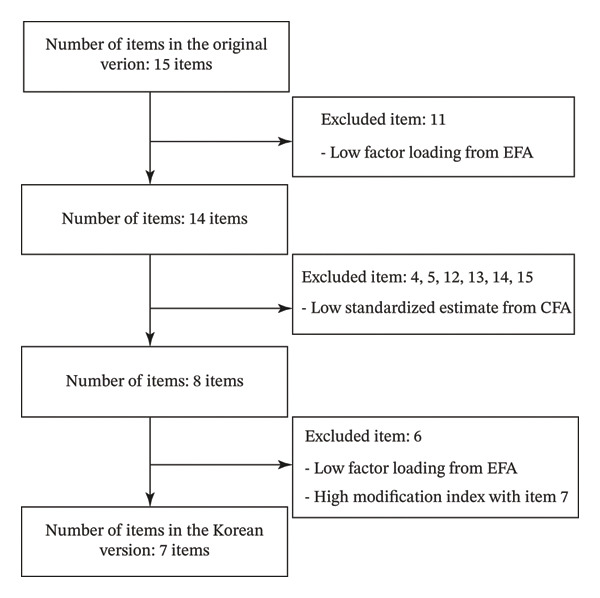
Item deletation process for the Korean version of the accumulated global changes from the professional bereavement scale.

Fifteen items were initially analyzed for the PBS‐AGC; however, Item 11 was excluded from the analysis because it had a factor loading of ≤ 0.5. With 14 items, after confirming the results of EFA, CFA was conducted. Item 13 was also excluded, as the standardized estimate (*β*) was 0.15, not meeting the standard value of ≥ 0.50 [21]. Owing to the same issue, five more items (i.e., Items 4, 5, 12, 14, and 15) were excluded from the analysis. With eight items (Items 1, 2, 3, 6, 7, 8, 9, and 10), the KMO was 0.64, and the results of Bartlett’s test of sphericity were χ^2^ = 362.02 (*p* < 0.001), with the communality for each item ranging from 0.59 to 0.77. However, Item 6 was removed due to having a low factor loading (< 0.5) and a high modification index (44.92 with item 7). Finally, seven items were used for EFA (Figure 1). The KMO value was 0.59, and the Bartlett’s test of sphericity was χ^2^ = 291.95 (*p* < 0.001). Three factors were extracted based on the eigenvalue and scree plot: all item communalities ranged from 0.59 to 0.77 (≥ 0.4), and factor loadings ranged from 0.75 to 0.87 (≥ 0.5). The eigenvalues for Factors 1, 2, and 3 were 2.03, 1.62, and 1.44, respectively. The explained variances were 28.96%, 23.16%, and 20.56% for Factors 1, 2, and 3, respectively, with a cumulative explained variance of 72.69%.

The original version has 15 items with 5 factors; however, the Korean version has 7 items with 3 factors. All two items of Factor 5 (better coping with patient deaths) and the one item from Factor 2 (more acceptance of limitations) in the original version were combined into Factor 1 in the Korean version, and all four items of Factor 1 (new insight) in the original version were divided into Factors 2 and 3 in the Korean version. Three factors from the Korean version were named as follows: “More Acceptance of Death,” “Cherishing the Moments,” and “New Insights into Life and Death.”

The model fitness from the CFA showed high fitness at all levels. The results of the specific model fit indices were as follows: χ^2^ = 19.51 (*p* = 0.052), χ^2^/df = 1.77, NFI = 0.95, TLI = 0.97, CFI = 0.98, RMSEA = 0.05, and sRMR = 0.03 (Table 5).

### 4.5. Convergent and Discriminant Validity

The standardized estimate (*β*) for the PBS–SBR ranged from 0.54 to 0.80, indicating that all items met the standard value of ≥ 0.50 [21]. The AVE was 0.42–0.57. Only Factor 3 satisfied the standard value of ≥ 0.50. However, the AVE was “more conservative”, and the appropriateness of convergent validity could be confirmed based on CR, even if it was < 0.5 [22]. Since the CR ranged from 0.73 to 0.88, the convergent validity of the Korean version was adequate. In addition, the HTMT ratio between the factors ranged from 0.42 to 0.82, which were below the recommended threshold of 0.85, confirming the adequate discriminant validity (Table 5).

Table 5 provides the results of CFA for the PBS–AGC. The standardized estimate (*β*), except for Item 7, was 0.59–0.90. The standardized estimate of Item 7 was 0.48; however, considering the theoretical importance of the item and the satisfactory psychometric properties of the construct, this item was retained. The AVE and the CR of Factors 1 and 3 were above the standard values of 0.50 and 0.60, respectively (AVE: 0.58 and CR: 0.79 for Factor 1; AVE: 0.60 and CR: 0.74 for Factor 3). The AVE and CR for Factor 2 were 0.45 and 0.62, respectively. Although the AVE was < 0.5, the CR was > 0.6 [22]; thus, the convergent validity of the Korean version was confirmed. Discriminant validity was confirmed as all the HTMT ratios were below 0.85 (Table 5).

### 4.6. Criterion Validity

The Pearson’s correlation coefficient between the PBS–SBR and burnout from the ProQOL showed a fairly positive correlation (*r* = 0.31, *p* < 0.001), confirming criterion validity. Similarly, the fair criterion validity of the PBS–AGC was verified by positive correlations with compassion satisfaction and secondary traumatic stress on the ProQOL (*r* = 0.40, *p* < 0.001; *r* = 0.29, *p* < 0.001, respectively).

## 5. Discussion

In this study, the PBS developed by Chen and Chow [12] was translated into Korean and administered to nurses to examine its validity and reliability. The Korean version of the PBS–SBR demonstrated adequate psychometric properties, confirming its applicability as a tool for various studies measuring professional bereavement. However, this study provided preliminary evidence for the validity and reliability of the Korean version of the PBS–AGC.

A notable difference was observed between the Korean version of the PBS developed in this study and the original scale in terms of factor structure. EFA and CFA were conducted to evaluate construct validity, and the PBS–SBR and PBS–AGC each yielded three factors. While the original instrument comprised four factors (“Frustration and Trauma,” “Guilt,” “Grief,” and “Being Moved”) for the PBS–SBR, this study reconstructed them into three factors (“Guilty and Frustration,” “Grief and Trauma,” and “Being Moved”). Some items from the original “Frustration and Trauma” factor and all items from the “Guilt” factor were integrated into a single factor. This restructuring may be attributed to linguistic and cultural differences in the expression and bereavement responses following patient death.

In particular, the emergence of “Being Moved” as a distinct and robust factor underscores the profound significance of relational reciprocity within the Korean nursing context. In Korean society, which deeply values “Jeong” (a unique form of emotional attachment) and interpersonal bonds, the gratitude and understanding expressed by bereaved families (Items 4 and 6) serve as more than mere professional feedback. As demonstrated in qualitative research on Korean nurses’ process of accepting patient deaths, nurses develop deep attentiveness through grief, moving through stages of being close by, acknowledging together, and accompanying, suggesting that emotional exchange is central to how Korean nurses process loss and reaffirm their professional identity [23]. Such exchanges function as a powerful “emotional buffer” that mitigates the trauma of patient death and fosters resilience. These relational characteristics reflect a culturally specific bereavement pattern that differentiates Korean nurses from those in more individualistic Western cultures, where bereavement is often processed as an internal, individual experience. Indeed, given that end‐of‐life care in Korean tertiary hospital settings is particularly intense and nurses are frequently excluded from key clinical decisions, relational bonds with bereaved families become an even more vital source of professional support and meaning [24].

Furthermore, while Chen and Chow [12] examined physicians and nurses working in urban hospitals in mainland China, the present study focused exclusively on nurses employed in advanced tertiary hospitals in Korea, which may explain the differences in perceptions and experiences of patient death. Consistent with this, a previous study identified discrepancies in perspectives between physicians and nurses regarding end‐of‐life care for patients in Korean healthcare settings [24]. Furthermore, whereas physicians often minimize their grief to maintain professional distance, nurses experience a more cumulative and relational form of bereavement due to their constant bedside care [15]. Such emotional burdens are particularly pronounced in the high‐acuity environment of Korean tertiary hospitals, where nurses frequently encounter intense terminal care demands and moral distress [25]. Consequently, validating the scale specifically within this population is essential to ensure its clinical relevance and sensitivity for a group at high risk of professional bereavement in Korea.

The original five‐factor, 15‐item structure for the PBS–AGC was reduced to a 3‐factor, 7‐item structure, named “More Acceptance of Death,” “Cherishing the Moments,” and “New Insights into Life and Death.” Compared to the original instrument, which was subdivided into five factors—“New Insights,” “More Acceptance of Limitations,” “More Death‐Related Anxiety,” “Less Influence from Patient Deaths,” and “Better Coping with Patient Deaths”—the number of factors and items was substantially reduced in the Korean version. Notably, all items corresponding to “More Acceptance of Limitations” and “More Death‐Related Anxiety” were removed. Although a direct comparison is difficult, this finding suggests that nurses working in tertiary hospitals in Korea perceive fewer negative aspects of professional bereavement experiences. These differences can be interpreted as reflecting variations in cultural contexts and healthcare environments. While the PBS–SBR measures immediate and transient physical and emotional responses to patient death, the PBS–AGC encompasses the accumulated changes in overall life resulting from repeated exposure to patient death inherent in professional roles [12]. Therefore, the cultural and healthcare system characteristics may have exerted an even greater influence on the observed differences. In a qualitative study of professional bereavement experiences among Chinese physicians and nurses, Chen et al. [26] reported that such experiences are shaped by workplace violence against professional caregivers, traditional Chinese medical ethics, strong death taboos, and inadequacies of the healthcare system. These findings suggest that, in contrast to the Korean cultural perspective, the pronounced sociocultural taboos of death in China may have influenced the observed results. Furthermore, differences in healthcare systems and institutional characteristics of the medical settings in which the participating healthcare professionals were employed may have also contributed to variations in the items and factor structures of the PBS. Accordingly, a further qualitative study conducted among nurses in mainland China demonstrated that attitudes toward death and end‐of‐life care can be delineated into personal and professional dimensions [27]. The personal dimension is largely shaped by traditional cultural and societal attitudes toward death and dying, whereas the professional dimension is consistent with the values of nursing and palliative care, highlighting the distinct dissonance between the two. Collectively, these findings indicate that bereavement experiences in the professional role of nurses are qualitatively different from those in family bereavement and are highly contingent on the healthcare environment and sociocultural context.

In the specific context of Korean tertiary hospitals, these environmental influences are characterized by a unique combination of high‐acuity demands and hierarchical constraints. Unlike physicians, who may minimize grief to maintain professional distance, nurses’ bereavement is inherently cumulative and relational, shaped by their continuous bedside presence [15]. However, Korean nurses in these settings often encounter intense moral distress as they are required to perform life‐sustaining treatments they perceive as futile, while remaining largely excluded from final medical decision‐making[25]. This lack of agency, combined with the burden of continuous care, has been shown to contribute to significantly higher psychological distress in nurses relative to other healthcare professionals [24].

In particular, the participants in this study were exposed to patient death at a mean frequency of 7.17 times per month. Such high‐frequency exposure could potentially foster what can be described as “emotional efficiency for professional survival,” a possible adaptive process through which nurses, upon repeated encounters with death, may progressively disengage from complex existential rumination in favor of more streamlined psychological responses. This suggested adaptive trajectory may account for why the accumulated changes captured in the Korean version of the PBS–AGC converge around acceptance of death and present‐focused engagement with life (Items 1, 3, 8, and 10), rather than the deeper existential questioning represented by the eliminated items. Consistent with this interpretation, prior research has demonstrated that ICU nurses with extensive exposure to patient deaths develop emotional numbing as a cumulative adaptive response [28], and that on‐the‐job experience enables nurses to compartmentalize grief and set emotional boundaries as a form of self‐preservation [29, 30]. Taken together, this pattern may be understood as a form of “psychological compartmentalization”—a professional adaptation strategy potentially shaped by the high‐pressure, high‐mortality environment of Korean tertiary hospitals.

In such a constrained clinical hierarchy, where overt behavioral changes or systemic adjustments are difficult for individual nurses to initiate, an “accumulated change” may manifest primarily through internal cognitive restructuring and existential integration rather than outward behavioral shifts. This could explain why the Korean version of the PBS–AGC was refined into a structure centered on internal acceptance and new insights into life and death—representing the most adaptive psychological mechanism available for professional survival within the high‐pressure environment of Korean tertiary hospitals.

Compared to previously employed instruments in the field of professional bereavement research, the Korean version of the PBS has several advantages. First, the Korean version validated in this study provides a tool that captures the characteristics of the professional bereavement experiences of nurses. In contrast, instruments commonly used in previous studies, such as the Grief Experience Inventory [9], Inventory of Complicated Grief [10], and Texas Revised Inventory of Grief [11], were originally developed to assess general loss experiences or family bereavement. Thus, their application to healthcare professionals has been limited by their inability to reflect the distinctive features of the professional context [12]. Moreover, this limitation has been repeatedly noted in recent studies. For example, Phillips et al. [29] described professional grief of healthcare professionals as being “hidden in plain sight,” underscoring the inadequacy of existing measures, while McGue et al. [31] similarly emphasized the need for appropriate tools to assess grief among oncology providers. In this context, the Korean version of the PBS is particularly significant as it is grounded in the original instrument developed by Chen and Chow [12] and validated specifically among nurses, thereby offering a measure that captures professional bereavement experiences distinct from general bereavement.

Second, in the context of the growing body of research on healthcare professionals’ grief following the COVID‐19 pandemic [4], this instrument holds particular significance, as it provides a culturally adapted tool for systematically evaluating professional bereavement within the Korean healthcare setting. Previous studies have highlighted the importance of such culturally adapted tools. For example, Singh et al. [32] reported model fit issues attributable to cultural differences when applying the Grief Support Healthcare Scale to Malaysian ICU nurses. Thus, the validation of the PBS in the Korean cultural context has substantial value. While the reduction in the number of items might be perceived as a compromise in scope, the high factor loadings and robust model fit suggest that the 7‐item PBS–AGC captures the theoretical core of the “AGC.” Rather than a loss of essential breadth, this refinement reflects an optimization toward theoretical potency, focusing on the internal cognitive restructuring most relevant to the Korean nursing context. This is consistent with findings by Kang et al. [25] and Choi et al. [24], which suggest that the lack of clinical agency among Korean nurses necessitates an internal, rather than external, adaptive process for professional survival. Therefore, the Korean version of the PBS–AGC does not merely offer a brief version of the original scale; instead, it provides a more culturally and professionally sensitive measurement by focusing on the existential integration that is most relevant to nurses at high risk of professional bereavement. By prioritizing these theoretically potent aspects, the Korean version of the PBS ensures greater clinical utility and sensitivity within the Korean healthcare landscape.

Third, the Korean version of the PBS demonstrates the strength of the comprehensiveness of its measurement dimensions. While previous instruments have predominantly focused on negative responses (e.g., grief, trauma, and complicated grief), the PBS, based on Chen and Chow [12], incorporates a factor structure that captures negative responses (PBS–SBR) and adaptive growth and change (PBS–AGC). This reflects the findings of Wilson [33] and Granek et al. [13], who emphasized that the bereavement experiences of healthcare professionals are not solely negative, but may provide opportunities for professional growth and the discovery of meaning. Thus, by assessing the negative aspects and adaptive growth and change, the Korean version of the PBS offers a foundation for developing strategies to support nurses, who, due to the nature of their profession, are repeatedly exposed to patient death, in adapting to and growing through these experiences.

The Korean version of the PBS validated in this study can contribute to nursing management in several ways. First, by systematically assessing nurses’ professional bereavement, nurse managers can enable the early identification of its negative impacts and the provision of appropriate interventions. Considering the findings of Liang and Wang [34], who demonstrated the moderating effect of professional grief on nurses’ turnover intentions, systematic assessment using this tool may further contribute to workforce retention. Second, as emphasized by Sharif et al. [35], understanding the relationship between grief and coping strategies is crucial. In this regard, the PBS offers a foundation for research exploring this relationship, particularly through the adaptive growth dimension of the PBS–AGC, which can inform the development of positive coping strategies. Third, nurse managers can design and evaluate grief support programs in healthcare institutions using the PBS. For instance, in line with the hospital‐based interventions proposed by Yazdan et al. [36], the PBS can serve as an objective measure for assessing changes before and after an intervention. Moreover, as highlighted in a meta‐synthesis by Feng et al. [37], the PBS can facilitate the evaluation of the effectiveness of various bereavement coping strategies. Finally, the Korean version of the PBS reflects the specific characteristics of the Korean healthcare environment because it is based on a tool developed and validated by Chen and Chow [12] in the Chinese cultural context and subsequently validated among Korean nurses. Moreover, it may contribute to a deeper understanding of professional bereavement in Asian cultural contexts, providing nurse managers with generalizable insights about professional bereavement across cultures.

## 6. Limitations

This study has some limitations. First, the sampling process did not intentionally account for participants’ clinical departments or the specific intensity of their prior bereavement experiences. Consequently, there was significant variability in the frequency of exposure to patient death among the participants. Future studies should employ more controlled or stratified sampling approaches to minimize this variability and allow for systematic comparisons. Specifically, it is necessary to investigate whether the nature and intensity of professional bereavement experiences differ across various hospital units and how these characteristics evolve depending on the clinical context. Second, while the current sample reflects the predominantly female demographic of the nursing profession in South Korea, future studies should explore potential differences in professional bereavement experiences among male nurses, given their steadily increasing representation within the nursing workforce. Furthermore, examining the influence of individual and professional characteristics on nurses’ reactions to patient bereavement could provide a deeper understanding of the phenomenon. Third, this study did not include cognitive interviewing with the target population during the translation process, which may have limited the depth of cultural adaptation. Therefore, future studies should utilize cognitive interviewing to ensure the instrument is precisely tailored to the unique cultural and linguistic nuances of the Korean nursing context. In addition, as several statistical values were marginal, they should be interpreted with caution. For example, the KMO value for the PBS–AGC was marginal, and thus, the identified factor structure should be considered preliminary, requiring further validation with a larger sample size. Cronbach’s *α* for the PBS–AGC indicated modest internal consistency. Although this may be acceptable for a newly developed or early‐stage instrument [17], further studies are required to confirm the revised scale’s reliability. Furthermore, the factor structure of the Korean version of the PBS, particularly the PBS–AGC, should be interpreted with caution. The identified structure was derived from substantial modifications to the original instrument and reflects nurses’ experiences in Korean tertiary hospitals. Consequently, replication and cross‐validation in independent samples across diverse clinical settings are needed to establish the validity, reliability, and generalizability of the revised scale. Despite these limitations, the Korean version of the PBS validated in this study holds significant value, as it provides a reliable tool for accurately assessing the professional bereavement experiences of Korean nurses and establishes a foundation for developing interventions to manage these experiences effectively.

## 7. Conclusion

In this study, a tool for assessing nurses’ professional bereavement was translated into Korean, and its reliability and validity were validated. Through a rigorous validation process, multiple aspects of its psychometric properties were confirmed, including content, construct, convergent, discriminant, and criterion validity. Reliability was further demonstrated through internal consistency (Cronbach’s *α*) and test–retest reliability, confirming the stability and dependability of the instrument. The development of this validated tool is significant because it provides a psychometrically robust instrument capable of measuring the unique grief experiences of healthcare professionals, distinct from familial bereavement measures, thereby laying an important foundation for advancing research on nurses’ professional bereavement.

## 8. Implications for Nursing Practice

The Korean version of the PBS was validated in this study and can be employed to systematically assess and monitor the professional bereavement of nurses, enabling early identification of those who experience significant grief responses following patient death. Such an assessment capacity allows for the development of targeted interventions and support programs tailored to the specific needs of healthcare professionals experiencing grief, which may, in turn, reduce burnout experienced by nurses, enhance job satisfaction, and improve overall well‐being. Furthermore, by accurately measuring professional bereavement, the tool can assist nurse managers in developing evidence‐based bereavement policies, ultimately contributing to the creation of a more supportive work environment that acknowledges and addresses the emotional burden experienced during the course of patient care.

## Author Contributions

Haeyoung Lee: conceptualization, methodology, formal analysis, writing–original draft, and writing–review and editing. Sun Joo Jang: conceptualization, methodology, formal analysis, writing–original draft, and writing–review and editing. Sophia Chung: conceptualization, methodology, formal analysis, writing–original draft, writing–review and editing, and supervision.

## Funding

This research received no specific grant.

## Conflicts of Interest

The authors declare no conflicts of interest.

## Data Availability

This study was a secondary data analysis. The data are available from the corresponding author of the parent study upon reasonable request. The data are not publicly available due to privacy and ethical restrictions.
